# PDLIM2 Repression: A Common Mechanism in Viral Lung Infection

**DOI:** 10.1101/2025.09.12.675949

**Published:** 2025-09-17

**Authors:** Feng Gao, Yongzheng W. Chen, Steven D. Shapiro, Gutian Xiao, Zhaoxia Qu

**Affiliations:** 1 Department of Molecular Microbiology and Immunology, Hastings Center for Pulmonary Research, Norris Comprehensive Cancer Center, University of Southern California Keck School of Medicine, Los Angeles, CA 90033, USA; 2 Bridge Undergraduate Science (BUGS) Jr. Program, University of Southern California, Los Angeles, CA 90033, USA; 3 Department of Medicine, University of Southern California Keck School of Medicine, Los Angeles, CA 90033, USA

**Keywords:** PDLIM2 repression, NF-kB, Viral Lung Infection, Influenza, SARS-CoV-2

## Abstract

**Background:**

PDLIM2, a PDZ-LIM domain-containing protein expressed highest in the lung and immune cells, serves as a unique tumor suppressor and immune modulator, mainly by turning off the activation of the master transcription factors NF-κB and STAT3. While its role in cancer is established, the involvement of PDLIM2 in viral infection remains unclear.

**Results:**

Here, we analyzed public gene expression data of blood leukocytes, bronchoalveolar lavage cells, and lung tissues from uninfected healthy humans and those infected with the respiratory virus SARS-CoV-2 or influenza. We found that PDLIM2 expression was repressed by viral infection, and notably, this repression correlated with the severity of infectious diseases. Consistently, the expression level of PDLIM2 was negatively associated with NF-κB and STAT3 activity across a diverse range of cell types, such as macrophages, monocytes, neutrophils, T cells, alveolar type 1 and 2 epithelial cells, airway epithelial cells, and fibroblasts. Accordingly, cells with low PDLIM2 expression exhibited aberrant activation of signaling pathways essential for cellular functions and immune responses.

**Conclusions:**

These findings highlight PDLIM2 repression as a common mechanism underlying human viral infectious diseases and suggest PDLIM2 as a potential biomarker and therapeutic target for disease prognosis, prevention, and treatment.

## Background

Lung infection is a leading cause of hospitalization and death in the United States and worldwide [1–3]. And it causes significantly higher morbidity and mortality in individuals with pre-existing conditions, such as lung cancer, chronic obstructive pulmonary disease (COPD), and interstitial lung disease (ILD)/pulmonary fibrosis (PF) [4–6]. In line with the high risk and high death rate of lung infection, unfortunately, our understanding of lung infectious disease, and especially its molecular link with lung cancer and other lung diseases, remains far from complete.

Recent studies identify the PDZ-LIM domain-containing protein PDLIM2 as a key molecular checkpoint in preventing lung cancer, COPD, and ILD/PF [7–11]. PDLIM2, also known as mystique or SLIM, is ubiquitously expressed under physiological conditions, with the highest level in the lung [12–14]. At the cellular level, it is expressed abundantly in various epithelial and immune cells, particularly lung epithelial cells and myeloid cells [7–11, 14, 15]. However, its expression is repressed in the lung of patients with lung cancer, COPD and/or ILD/PF, and this repression is associated with disease severity and poor prognosis and survival [7–10]. In mice, global deletion or selective obliteration of PDLIM2 from lung epithelial cells or myeloid cells renders animals highly susceptible to spontaneous and induced lung tumors as well as to lung injury and death by the endotoxin lipopolysaccharide (LPS) [7, 8, 11]. Notably, PDLIM2 restoration by nano-delivery of exogenous PDLIM2 (nanoPDLIM2) or pharmacological induction of endogenous PDLIM2 shows promising therapeutic activities and improves the efficacy of chemotherapy and immunotherapy in the mouse models of lung cancer [7, 8, 10, 15].

PDLIM2 exerts its tumor suppressor role and immune regulation effect through multiple mechanisms [16–19]. The major one is to terminate the activation of nuclear factor kappaB (NF-κB) and signal transducer and activator of transcription 3 (STAT3) by promoting ubiquitination and proteasomal degradation of activated RelA (the prototypical NF-κB member, also known as p65) and STAT3 in the nucleus [7–10, 20–26]. NF-κB and STAT3 are master transcription factors that control inflammation, metabolic reprogramming, and cell survival; and their persistent activation plays causative roles in the pathogenesis of various human diseases, including lung cancer, COPD, ILD/PF, and infections [7–10, 27–39]. More recent mechanistic studies show that instead of being claimed before as a ubiquitin ligase (E3), PDLIM2 acts as a novel ubiquitin ligase enhancer (E5) to stabilize and chaperone the E3 SCF^β-TrCP^ to nuclear RelA, ubiquitinating it for proteasomal degradation [14, 26, 40, 41].

In this manuscript, we examined the possible involvement and clinical relevance of PDLIM2 in lung viral infection. We exploited public gene microarray and next-generation sequencing (NGS) data of peripheral blood leukocytes, bronchoalveolar lavage (BAL) cells, and lung tissues from uninfected ‘healthy’ humans and those infected with the respiratory virus severe acute respiratory syndrome coronavirus 2 (SARS-CoV-2) or influenza virus (IV). SARS-CoV-2 and IV are respectively the etiological agents of coronavirus disease 2019 (COVID-19) and influenza (commonly known as the flu), two of the most common respiratory illnesses that are highly contagious and often cause severe lung injury and death, particularly among vulnerable populations [1–3].

## Methods

### Data source and selection

All data were from the public Gene Expression Omnibus (GEO) repository that contains various microarray, bulk RNA-sequencing (RNA-seq), and single-cell RNA-seq (scRNA-seq) data. A search for multiomics data from human patients with viral lung infections yielded a list of datasets on IV and SARS-CoV-2 infections. The generated datasets were subjected to routine quality control analysis and PDLIM2 expression examination. Those with good intrinsic quality and comprehensive representation of cell responses were selected, and the samples/cells that were PDLIM2-positive were then processed with the analysis pipelines described below. GEO datasets for the final analyses are listed in Supplementary Table 1. The publications originally associated with these datasets were also listed [42–48].

### Gene microarray and bulk RNA-seq data analysis pipeline

These data were analyzed in RStudio in the way GEO2R performs. GEO2R, an online tool on the NCBI GEO website, offers data that has undergone alignment, quantification, and normalization processes by the original submitters or by NCBI's own pipelines, and allows for differential gene expression analysis of microarray and, in a beta version, bulk RNA-seq data. In brief, after a GEO Series was imported into RStudio, experimental groups (e.g., disease vs. control) were defined, and samples were assigned to these groups. Gene expression data of PDLIM2 and other genes were fetched along with their metadata. Statistical packages like Limma for microarray data and DESeq2 for RNA-seq (using NCBI-computed raw counts) were used to identify genes significantly altered between the defined groups. Results were then visualized with plots and heatmaps. The R scripts for the analysis were stored on GitHub (https://github.com/Xiao-Qu-Lab/PDLIM2-in-lung-viral-infection/blob/main/GSE101702.R; and https://github.com/Xiao-Qu-Lab/PDLIM2-in-lung-viral-infection/blob/main/GSE157103.R).

### Single-cell RNA-seq data analysis pipeline

These data were analyzed in Seurat, a popular R package for scRNA-seq data analysis. As scRNA-seq data in NCBI repository were deposited in variable formats and stages, our analysis always started with the processed data if available, and if not, a new analysis was carried out to recapitulate the paper’s results with the settings as defined in the paper’s methods. A full typical workflow involves: 1) Importing gene expression matrix and metadata to Seurat and quality control (QC) to filter low-quality cells. 2) Normalization and scaling to account for technical variations. 3) Dimensionality reduction (e.g., PCA, UMAP/t-SNE) to visualize cell relationships in lower dimensions. 4) Clustering to group cells with similar gene expression profiles, identifying distinct cell populations. 5) Marker gene identification to find genes uniquely expressed in each cluster, aiding cell type annotation. 6) Cell assignments to disease and control groups, with the former further divided into 2 disease subgroups based on the levels of the expression of PDLIM2. 7) Performing differential gene expression with the Wilcoxon Rank Sum test among the groups defined above. 8) Exporting gene expression data of PDLIM2 and other gene groups and differentially expressed genes (DEGs) to the local disk. 9) Gene expression data were visualized with plots and heatmaps, and DEGs were imported to Qiagen ingenuity pathway analysis (IPA) for pathway and functional analyses. The R scripts for the analysis were stored on GitHub (https://github.com/Xiao-Qu-Lab/PDLIM2-in-lung-viral-infection/blob/main/GSE243629.R; https://github.com/Xiao-Qu-Lab/PDLIM2-in-lung-viral-infection/blob/main/GSE145926.R; and https://github.com/Xiao-Qu-Lab/PDLIM2-in-lung-viral-infection/blob/main/GSE171524.R).

### Pathway activation score

The component and target genes of a specific signaling pathway were collected from Qiagen and Reactome, and other online resources, and their expressions were summed up and used as the activation score for the pathway. The source and the list of the genes for the pathways studied can be found in the R scripts stored on the GitHub above.

### PDLIM2 level-dependent pathway and function analysis

Besides the direct comparison between the disease and the control, samples from the disease were subset into PDLIM2-low and -high groups using the median as the divider, and differential gene expression was performed from their respective comparison to the control. The DEGs derived were imported into Qiagen IPA for pathway and functional analyses. Comparison analysis was done between PDLIM2 low and high conditions, and the differential activation of the pathway with significant P-values from both conditions was calculated as z-score deviation between them.

### Statistics

Student’s t-test (2-tailed, unpaired) and ordinary 1-way ANOVA were used to assess the significance of differences between two groups and among groups of more than two, respectively [49–51]. Bars on a box plot represent the minimum, lower quartile, median, upper quartile, and maximum, respectively. The Correlation Coefficient (R) for the trendline was calculated by dividing the covariance of the x and y axis variables by the product of their standard deviations. The P-value in IPA pathway analysis was calculated using Fisher's Exact Test. The z-score in IPA pathway analysis was calculated by comparing the observed gene expression changes in a dataset to the expected directional changes stored in the knowledge base, providing a statistical measure for the predicted activation or inhibition of a pathway. P-values less than 0.05 were considered statistically.

### Data and code access

All R scripts used for analysis were stored on GitHub (https://github.com/Xiao-Qu-Lab/PDLIM2-in-lung-viral-infection) and are freely accessible to the public.

## Results

### PDLIM2 repression by lung viral infection and its association with disease severity

**Fig. 1:**
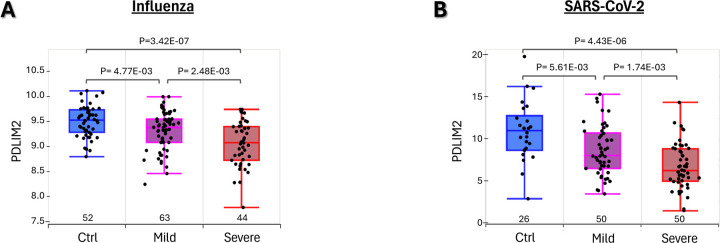
PDLIM2 repression by lung viral infection and its association with disease severity. (A) Gene microarray data analysis showing a negative association of PDLIM2 repression in human peripheral blood leukocytes with influenza severity. (B) Bulk RNA-seq data analysis showing a negative association of PDLIM2 repression in human peripheral blood leukocytes with COVID-19 severity. Sample numbers (N) are listed above the x-axis. Each data point represents one patient. Data is presented as box plots. P-values are obtained from ANOVA or Student’s *t* test.

We first analyzed human peripheral blood leukocyte gene microarray data on influenza and bulk RNA-seq data on COVID-19, because they may be used as a noninvasive and easy-to-perform assay for the prognosis of those infectious diseases. As expected, PDLIM2 was easily detected in the blood leukocytes from uninfected healthy people by either microarray (Fig. 1A) or bulk RNA-seq (Fig. 1B). However, its expression was repressed in patients with influenza or COVID-19. Notably, the extent of PDLIM2 repression was associated with increased severity in both diseases.

### Association of PDLIM2 repression with pathological activation and death of neutrophils

Following the promising results, we analyzed scRNA-seq data to examine PDLIM2 expression in peripheral neutrophils, as neutrophils are the most abundant leukocytes in human blood and serve as the first line of defense against infection. In response to lung infection, they move quickly and exponentially to the lung to kill invading pathogens and drive inflammation via several major related mechanisms, including phagocytosis, degranulation, oxidative burst, and release of neutrophil extracellular traps (NETs), a process called NETosis. They also secrete pro-inflammatory cytokines and chemokines, promoting inflammation. After pathogen clearance, neutrophils produce anti-inflammatory cytokines and undergo non-inflammatory apoptosis to be removed mainly by macrophages, leading to the resolution of inflammation and the restoration of tissue homeostasis. Dysregulation of those functions of neutrophils damages lung tissues and promotes disease severity (Supplementary Table 2).

**Fig. 2:**
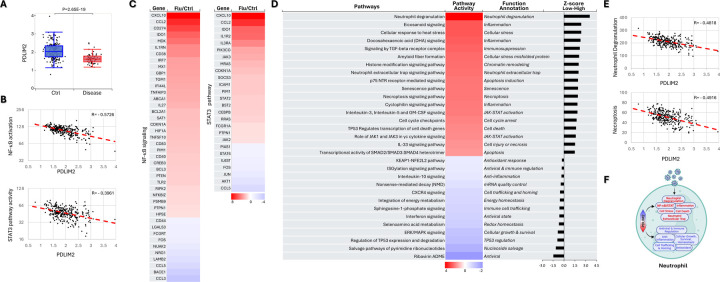
Association of PDLIM2 repression with pathological activation and death of neutrophils. (A) scRNA-seq data analysis showing PDLIM2 repression in the blood neutrophils of influenza patients. Data is presented as box plots. P-values are obtained from Student’s *t* test. (B) Gene and pathway correlation showing negative associations of PDLIM2 expression with activations of the NF-κB and STAT3 signaling pathways in human blood neutrophils. (C) Heatmap showing differential expression of NF-κB and STAT3 target genes in the blood neutrophils of influenza patients in comparison to uninfected ‘healthy’ controls. (D) Heatmap showing differential activation of signaling pathways in PDLIM2-low vs. -high expressing blood neutrophils in influenza patients. (E) Gene and pathway correlation showing negative associations of PDLIM2 expression with the activation of the neutrophil degranulation and necroptosis signaling pathways in human blood neutrophils. (F) Cartoon summary listing the main signaling pathways differentially activated in PDLIM2-low vs. -high expressing blood neutrophils in influenza patients.

Indeed, PDLIM2 expression was significantly lower in neutrophils from influenza patients in comparison to those from uninfected healthy people (Fig. 2A). Consistent with its known functions, PDLIM2’s expression levels were negatively associated with the activation scores of NF-κB and STAT3 in neutrophils (Fig. 2B). Accordingly, numerous target genes of NF-κB and STAT3 were predominantly more activated in patients’ cells (Fig. 2C). Besides NF-κB and STAT3, many other signaling pathways were detrimentally activated in patients’ neutrophils (Supplementary Fig. 1). Some of them were deregulated also in a manner dependent on PDLIM2 repression, as evidenced by their different activations in the PDLIM2-low versus -high cells of patients and by the association between the activities of these signaling pathways and PDLIM2 expression level (Fig. 4D–4F). Notably, several pathways propelling inflammatory responses, including neutrophil degranulation, necroptosis, NETosis, cellular stress, senescence, and cyclophilin signaling pathways, were increased, whereas those resolving inflammation, such as interleukin-10 (IL-10) signaling were decreased in PDLIM2-low cells. The antiviral interferon (IFN) signaling pathway and the master CXCR4 bone marrow retention signaling of neutrophils were also decreased in PDLIM2-low cells.

### Association of PDLIM2 repression with hyper-activation of monocytes

Upon lung infection, peripheral monocytes, like neutrophils, rapidly migrate to the lung, assisting viral clearance mainly through differentiating into highly inflammatory macrophages and presenting viral antigens to boost adaptive immunity. Monocytes also produce pro-inflammatory cytokines and substances to enhance inflammation. Not surprisingly, overactivation of monocytes leads to excessive inflammation and lung damage, and pathology (Supplementary Table 3).

**Fig. 3:**
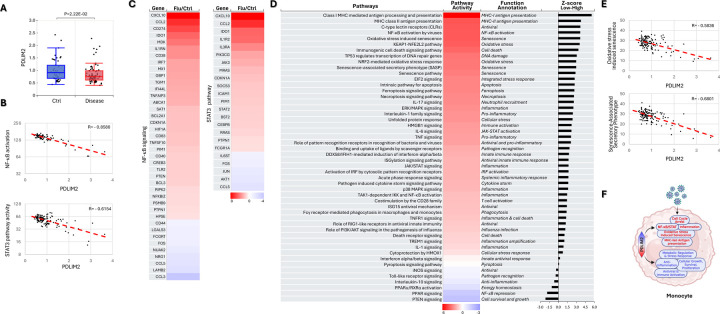
Association of PDLIM2 repression with hyper-activation of monocytes. (A) scRNA-seq data analysis showing PDLIM2 repression in the blood monocytes of influenza patients. P-values are obtained from Student’s *t* test. (B) Gene and pathway correlation showing negative associations of PDLIM2 expression with activations of the NF-κB and STAT3 signaling pathways in human blood monocytes. (C) Heatmap showing differential expression of NF-κB and STAT3 target genes in the blood monocytes of influenza patients in comparison to uninfected ‘healthy’ controls. (D) Heatmap showing differential activation of signaling pathways in PDLIM2-low vs. -high expressing blood monocytes in influenza patients. (E) Gene and pathway correlation showing negative associations of PDLIM2 expression with the activation of the oxidative stress and SASP signaling pathways in human blood monocytes. (F) Cartoon summary listing the main signaling pathways differentially activated in PDLIM2-low vs. -high expressing blood monocytes in influenza.

Same as in neutrophils, PDLIM2 expression was repressed in monocytes from influenza patients compared to those from uninfected healthy humans; and this repression was associated with ultra-activation of NF-κB and STAT3 (Fig. 3A–3C). Numerous other signaling pathways critical for monocytes as the contributors of inflammatory responses were also overwhelmingly activated in patients (Supplementary Fig. 2). Like NF-κB and STAT3, many of these signaling pathways were over-activated in a manner depending on PDLIM2 repression, such as oxidative stress, senescence-associated secretory phenotype (SASP), high mobility group box 1 (HMGB1), triggering receptor expressed on myeloid cells 1 (TREM1), and pathogen-induced cytokine storm signaling pathways (Fig. 3D–3F).

### Association of PDLIM2 repression with pathogenic activation of lung macrophages

Macrophages, the primary phagocytes, are the most abundant immune cells within the lung. Particularly, those residing in and patrolling the alveoli serve as the first immune cell responder to lung infection, killing inhaled pathogens and turning on inflammation and resolving it to promote lung tissue healing after pathogen clearance. Dysregulation in this functional switch of those key sentinels leads to uncontrolled inflammation and causes severe lung damage and often death (Supplementary Table 4).

**Fig. 4:**
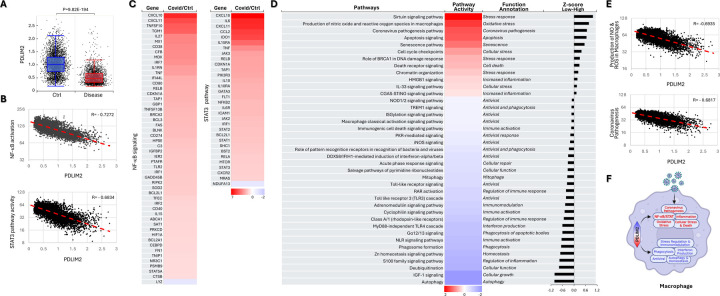
Association of PDLIM2 repression with pathogenic activation of lung macrophages. (A) scRNA-seq data analysis showing PDLIM2 repression in the lung macrophages of COVID-19 patients. P-value was obtained from Student’s *t* test. (B) Gene and pathway correlation showing negative associations of PDLIM2 expression with activations of the NF-κB and STAT3 signaling pathways in human lung macrophages. (C) Heatmap showing differential expression of NF-κB and STAT3 target genes in the lung macrophages of COVID-19 patients in comparison to uninfected controls. (D) Heatmap showing differential activation of signaling pathways in PDLIM2-low vs. -high expressing lung macrophages in COVID patients. (E) Gene and pathway correlation showing negative associations of PDLIM2 expression with the activation of the production of NO & ROS and coronavirus pathogenesis signaling pathway in human lung macrophages. (F) Cartoon summary listing the main signaling pathways differentially activated in PDLIM2-low vs. -high expressing lung macrophages in COVID-19 patients.

Analysis of the scRNA-seq data of BAL cells showed that many signaling pathways were activated differently in the lung macrophages from COVID-19 patients compared to those from uninfected controls (Supplementary Fig. 3). Importantly, we identified a drastic PDLIM2 repression and a strong negative association of PDLIM2 expression with NF-κB and STAT3 activation in these culprit cells of the fatal pathogenesis (Fig. 4A–4C). In addition to NF-κB and STAT3, several other pro-inflammatory signaling pathways, such as oxidative stress, senescence, HMGB1, and coronavirus pathogenesis pathways, were hyper-activated in a PDLIM2 repression-dependent manner (Fig. 4D–4F). On the contrary, those involved in viral clearance and inflammation resolution, including phagocytosis, autophagy, insulin-like growth factor (IGF), and S100 family signaling, were hypo-activated, which also depended on PDLIM2 repression. Of note, autophagy also serves as an intrinsic checkpoint restricting NF-κB activities [52–55].

### Association of PDLIM2 repression with T-cell exhaustion and senescence

During lung infection and especially when myeloid cells (macrophages and neutrophils) are insufficient to control it, T cells are activated and migrate into the lung where they eliminate infected cells and release cytokines that help control the infection. Similarly, T cells may also contribute to lung inflammation and injury, including those caused by viruses like SARS-CoV-2 (Supplementary Table 5 and Supplementary Fig. 4). Same as myeloid cells, T cells in COVID-19 patients expressed PDLIM2 at a much lower level and had high NF-κB and STAT3 activation (Fig. 4A–4D). On the other hand, Rho GDP-dissociation inhibitor (RhoGDI) signaling, which plays essential roles in various cellular functions, including T cell activation and migration, was lower in T cells with low PDLIM2 expression in comparison to those with high PDLIM2 expression. Remarkably, T cells with low PDLIM2 expression were more exhausted and with an increased SASP.

### Association of PDLIM2 repression with viral endocytic entry and immunogenic cell deaths of alveolar type 2 epithelial cells

Alveolar type 2 (AT2) cells, one of the two major types of epithelial cells lining the pulmonary alveoli, are the primary source of pulmonary surfactants and adult tissue stem/progenitor cells, thereby critical for respiratory homeostasis and lung tissue repair after injury. However, they are also the main targets of lung infection, and their dysfunction and damage may lead to various lung conditions and often death. Despite being non-immune cells, AT2 cells possess unique immunologic properties and act as active participants in both the innate and adaptive immune responses, influencing the severity and outcome of diseases like COVID-19 (Supplementary Table 6).

**Fig. 6:**
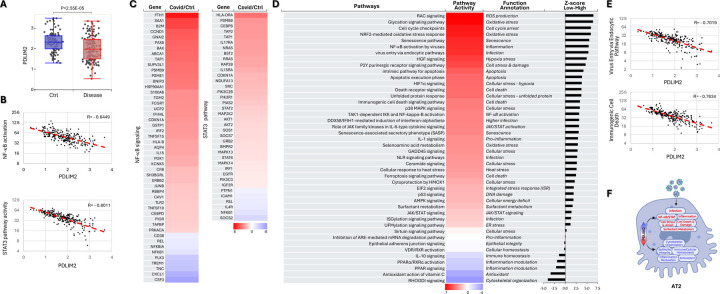
Association of PDLIM2 repression with viral endocytic entry and immunogenic cell deaths of AT2 cells. (A) scRNA-seq data analysis showing PDLIM2 repression in the AT2 cells of COVID-19 patients. P-value was obtained from Student’s *t* test. (B) Gene and pathway correlation showing negative associations of PDLIM2 expression with activations of the NF-κB and STAT3 signaling pathways in human AT2 cells. (C) Heatmap showing differential expression of NF-κB and STAT3 target genes in the AT2 cells of COVID-19 patients in comparison to uninfected ‘healthy’ controls. (D) Heatmap showing differential activation of signaling pathways in PDLIM2-low vs. -high expressing AT2 cells in COVID-19 patients. (E) Gene and pathway correlation showing negative associations of PDLIM2 expression with the activation of the endocytic viral entry and immunological cell death signaling pathways in human AT2 cells. (F) Cartoon summary listing the main signaling pathways differentially activated in PDLIM2-low vs. -high expressing AT2 cells in COVID-19 patients.

Analysis of scRNA-seq data of human lung tissues revealed that a plethora of signaling pathways were altered within the AT2 cells of COVID-19 patients (Supplementary Fig. 5). Many of them and particularly those involved in the fundamental activities of AT2 cells were deregulated in association with PDLIM2 repression, such as NF-κB, STAT3, surfactant metabolism, oxidative stress and immune regulation pathways (Fig. 6A–6D). Intriguingly, endocytic viral entry, senescence, and immunological cell death pathways were markedly increased, whereas RhoGDI, peroxisome proliferator-activated receptor (PPAR), and IL-10 signaling pathways were decreased in AT2 cells in which PDLIM2 was repressed (Fig.6D–6F). Note, PPAR signaling, like RhoGDI signaling, is involved in the cell repair, maintenance, and differentiation of AT2 and many other cells. PPAR signaling can also repress NF-κB.

### Association of PDLIM2 repression with alveolar type 1 epithelial cell damage

Alveolar type 1 (AT1) cells, the major type of alveolar epithelial cells other than AT2 cells, are responsible for gas exchange in the lung. They also play a significant role in lung immunity, interacting directly with immune cells and releasing immune mediators like cytokines and chemokines. AT1 cell dysfunction or damage is a major factor in various lung conditions, including infectious diseases like COVID-19, and often leads to severe outcomes and death (Supplementary Table 7 and Supplementary Fig. 6).

**Fig. 7:**
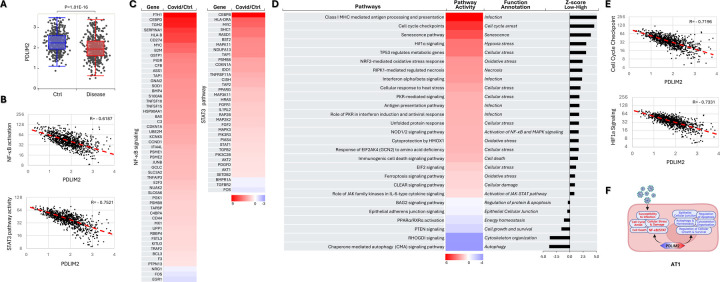
Association of PDLIM2 repression with AT1 cell damage. (A) scRNA-seq data analysis showing PDLIM2 repression in the AT1 cells of COVID-19 patients. P-value was obtained from Student’s *t* test. (B) Gene and pathway correlation showing negative associations of PDLIM2 expression with activations of the NF-κB and STAT3 signaling pathways in human AT1 cells. (C) Heatmap showing differential expression of NF-κB and STAT3 target genes in the AT1 cells of COVID-19 patients in comparison to uninfected ‘healthy’ controls. (D) Heatmap showing differential activation of signaling pathways in PDLIM2-low vs. -high expressing AT1 cells in COVID-19 patients. (E) Gene and pathway correlation showing negative associations of PDLIM2 expression with the activation of the HIF and cell cycle checkpoints signaling pathways in human AT1 cells. (F) Cartoon summary listing the main signaling pathways differentially activated in PDLIM2-low vs. -high expressing AT1 cells in COVID-19 patients.

Like AT2 cells, AT1 cells in COVID-19 patients had a PDLIM2 repression-dependent hyper-activation of NF-κB and STAT3 (Fig. 7A–7D). Other over-activated signaling pathways dependent on PDLIM2 repression included those involved in senescence, cell cycle arrest, oxidative and cellular stress, and immunological cell death (Fig. 7D–7F). Interestingly, RhoGDI signaling pathway and chaperone-mediated autophagy (CMA), another signaling vital for AT1 cell repair, maintenance, and function, were decreased significantly in patients’ AT1 cells with low PDLIM2 expression.

### Association of PDLIM2 repression with inflammatory activation of airway epithelial cells

**Fig. 8:**
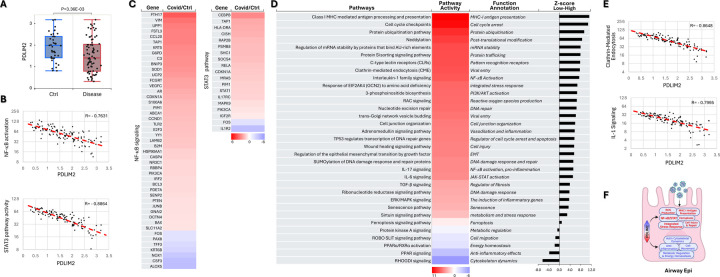
Association of PDLIM2 repression with AEC inflammatory activation. (A) scRNA-seq data analysis showing PDLIM2 repression in the AECs of COVID-19 patients. P-value was obtained from Student’s *t* test. (B) Gene and pathway correlation showing negative associations of PDLIM2 expression with activations of the NF-κB and STAT3 signaling pathways in human ACEs. (C) Heatmap showing differential expression of NF-κB and STAT3 target genes in the AT1 cells of COVID-19 patients in comparison to uninfected controls. (D) Heatmap showing differential activation of signaling pathways in PDLIM2-low vs. -high expressing AECs in COVID-19 patients. (E) Gene and pathway correlation showing negative associations of PDLIM2 expression with the activation of the IL-1 and clathrin-mediated endocytosis signaling pathways in human AECs. (F) Cartoon summary listing the main signaling pathways differentially activated in PDLIM2-low vs. -high expressing AECs in COVID-19 patients.

Airway epithelial cells (AECs) are the first cells in the lung to encounter inhaled substances like viruses. They form a physical barrier and participate actively in immune responses by releasing chemokines, cytokines, and defensins. However, AECs are also the primary targets for respiratory viruses, and their dysfunction or damage significantly contributes to lung injury and disease progression, including in the context of COVID-19 (Supplementary Table 8 and Supplementary Fig. 7). Mechanistically, we found that PDLIM2 was repressed in the AECs of COVID-19 patients, associating with increased NF-κB, STAT3, cell cycle checkpoints, senescence, clathrin-mediated endocytosis (CME), and pro-inflammatory cytokines but decreased RhoGDI and PPAR signaling pathways (Fig. 8).

### Association of PDLIM2 repression with pro-inflammatory and pro-fibrotic activation of lung fibroblasts

**Fig. 9:**
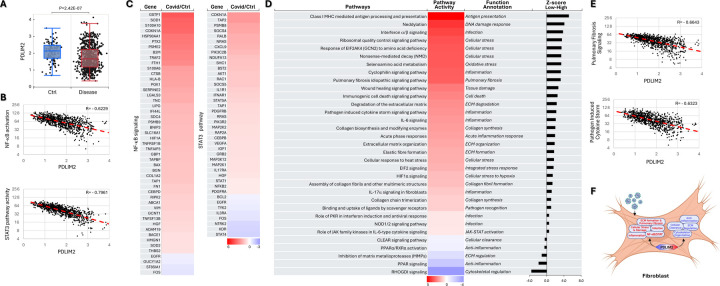
Association of PDLIM2 repression with pro-inflammatory and pro-fibrotic activation of lung fibroblasts. (A) scRNA-seq data analysis showing PDLIM2 repression in the lung fibroblasts of COVID-19 patients. P-value was obtained from Student’s *t* test. (B) Gene and pathway correlation showing negative associations of PDLIM2 expression with activations of the NF-κB and STAT3 signaling pathways in human lung fibroblasts. (C) Heatmap showing differential expression of NF-κB and STAT3 target genes in the lung fibroblasts of COVID-19 patients in comparison to uninfected controls. (D) Heatmap showing differential activation of signaling pathways in PDLIM2-low vs. -high expressing lung fibroblasts in COVID-19 patients. (E) Gene and pathway correlation showing negative associations of PDLIM2 expression with the activation of the pulmonary fibrosis and pathogen-induced cytokine storm signaling pathways in human lung fibroblasts. (F) Cartoon summary listing the main signaling pathways differentially activated in PDLIM2-low vs. -high expressing lung fibroblasts in COVID-19 patients.

Lung fibroblasts, while not being primary targets of lung infections like lung epithelial cells, play a critical role in both lung health and disease. They actively modulate immune responses and can contribute to tissue damage and fibrosis through interactions with other lung cells, particularly epithelial and immune cells. In response to inflammatory signals like those triggered by SARS-CoV-2 infection, they may produce excessive cytokines, chemokines, proteases and extracellular matrices (ECMs) and differentiate into myofibroblasts, potentially leading to pulmonary fibrosis and long-term lung damage (Supplementary Table 9 and Supplementary Fig. 8). The pathological activation of lung fibroblasts in COVID-19 patients involved intrinsic PDLIM2 repression (Fig. 9A), Because the NF-κB, STAT3, cellular stress, pulmonary fibrosis, pathogen-induced cytokine storm. immunogenic cell death and cyclophilin signaling pathways were significantly higher, while the RhoGDI, PPAR and inhibition of matrix metalloproteases (MMPs) signaling pathways were lower in PDLIM2-low lung fibroblasts of COVID-19 patients (Fig. 9B–9F).

## Discussion

Our study highlights both the importance and the clinical applicability of PDLIM2 expression as a biomarker to predict the development and progression of infectious diseases after lung infections by viruses like IV and SARS-CoV-2. The inverse association of PDLIM2 expression in peripheral blood leukocytes with disease severity in respiratory virus-infected humans provides a noninvasive and cost-effective method for diagnosis and prognosis. Furthermore, PDLIM2 repression in BAL cells offers a minimally invasive alternative, while examining PDLIM2 in lung tissues might be a costly and invasive one.

Our study also identifies PDLIM2 repression as a potential new mechanism driving infectious disease onset and progression in virus-infected people, deepening our understanding of viral infection and infectious diseases and revealing new pathophysiological roles of PDLIM2, more importantly, holding significant therapeutic implications. PDLIM2 is repressed in various immune cells and lung structural and functional cells, including lung macrophages, monocytes, neutrophils, T cells, AT1 cells, AT2 cells, AECs, and fibroblasts. Importantly, PDLIM2 repression is associated with aberrant activations of numerous common or cell type-specific signaling pathways, e.g., downregulation of the IL-10, RhoGDI, and PPAR signaling pathways in most cell types, whereas excessive activation of NF-κB, STAT3, cellular stress, senescence, immunogenic cell death, and pathogen-induced cytokine storm signaling pathways in all or most of those cell types, NETosis in neutrophils, and pulmonary fibrosis in fibroblasts. Remarkably, most associates of PDLIM2 are newly identified, with the exceptions of NF-κB and STAT3, which are known downstream targets and mediators of PDLIM2. PDLIM2 likely regulates these new associates directly or indirectly via NF-κB and STAT3, given their frequent crosstalk and shared signaling molecules and target genes. Vice versa, PDLIM2 repression is attributed to oxidative stress-related signaling pathways activated by lung infection, since PDLIM2 expression could be repressed by the reactive oxygen species (ROS)-activated transcription repressor BACH1 [15]. These findings provide mechanistic insights into how PDLIM2 serves as the central nexus of the complex molecular and cellular signaling network crucial for lung health and disease.

Of note, PDLIM2 exhibits multifaceted regulatory effects on signaling pathways, with both positive and negative outcomes depending on cell type and microenvironment. For instance, PDLIM2 repression enhances CXCR4 signaling in monocytes while diminishing it in neutrophils. Similarly, cyclophilin signaling is upregulated in PDLIM2-low neutrophils and fibroblasts but downregulated in PDLIM2-low macrophages. Moreover, while the overall effect of PDLIM2 repression and cell activation in infectious diseases is pathological, they may simultaneously contribute to antiviral activity. Specifically, an increase in antiviral antigen presentation, IFN, and ISGylation signaling pathways is detected across all patient cell types. Their increase in monocytes, AT1 cells, AECs, and fibroblasts involves PDLIM2 repression, whereas PDLIM2 repression attenuates IFN and ISGylation signaling in neutrophils. However, the detected antiviral activity is dominated by the pathological activation of other signaling pathways, especially when the primary target cells of the antiviral activity are defective (e.g., T cells are exhausted, and macrophages are deficient in phagocytosis). In fact, under this situation, excessively high or sustained levels of IFNs mainly contribute to the detrimental inflammatory response, tissue damage, and disease pathogenesis.

Our study has limitations, including its retrospective design, the heterogeneity of the study population, and the lack of detailed information on patients’ demographics and medical history. Additionally, we did not include all cell types, excluding B cells and dendritic cells (DCs) and natural killer (NK) cells, and did not delve into subtypes of cells, particularly for T cells and AECs, which consist of multiple subtypes. Furthermore, the gene expression thresholds were derived internally from this dataset, which may impact generalizability. Despite these limitations, our study presents valuable biomarker discovery, underscoring the need for future prospective studies to validate the prognostic value of PDLIM2 expression in viral infectious diseases, especially influenza and COVID-19.

## Conclusions

In summary, our study demonstrates that low PDLIM2 expression in the context of influenza and COVID-19 correlates with unfavorable molecular and cellular profiles of pro-inflammation and pro-fibrosis and cell injury, and is associated with increased disease severity. These findings establish PDLIM2 as a potential prognostic marker for predicting disease development and progression in individuals infected with viruses like IV and SARS-CoV-2. Furthermore, they highlight the potential of PDLIM2 as a therapeutic target for the prevention and treatment of infectious diseases, especially given the high therapeutic efficacy and safety profile of nanoPDLIM2 in lung cancer models. Considering that PDLIM2 expression is affected in a larger range of cell types in infectious diseases compared to lung cancer, nanoPDLIM2 or other PDLIM2-targeted therapies may offer even better efficacy and safety for patients with infectious diseases.

## Supplementary Material

1 **Fig. S1**: Top pathways altered in patient neutrophils.
**Fig. S2**: Top pathways altered in patient monocytes.
**Fig. S3**: Top pathways altered in patient macrophages.
**Fig. S4**: Top pathways altered in patient T cells.
**Fig. S5**: Top pathways altered in patient AT2 cells.
**Fig. S6**: Top pathways altered in patient AT1 cells.
**Fig. S7**: Top pathways altered in patient airway epithelial cells.
**Fig. S8**: Top pathways altered in patient fibroblasts.
**Table S1:** List of GEO datasets analyzed in the paper.
**Table S2:** Main differences of neutrophil-related pathways and biological processes in patients with mild and severe conditions by lung viral infection.
**Table S3:** Main differences of monocyte-related pathways and biological processes in patients with mild and severe conditions by lung viral infection.
**Table S4:** Main differences of macrophage-related pathways and biological processes in patients with mild and severe conditions by lung viral infection.
**Table S5:** Main differences of T-cell-related pathways and biological processes in patients with mild and severe conditions by lung viral infection.
**Table S6:** Main differences of AT2 cell-related pathways and biological processes in patients with mild and severe conditions by lung viral infection.
**Table S7:** Main differences of AT1 cell-related pathways and biological processes in patients with mild and severe conditions by lung viral infection.
**Table S8:** Main differences of AEC-related pathways and biological processes in patients with mild and severe conditions by lung viral infection.
**Table S9:** Main differences of lung fibroblast-related pathways and biological processes in patients with mild and severe conditions by lung viral infection.

## Figures and Tables

**Fig. 5:**
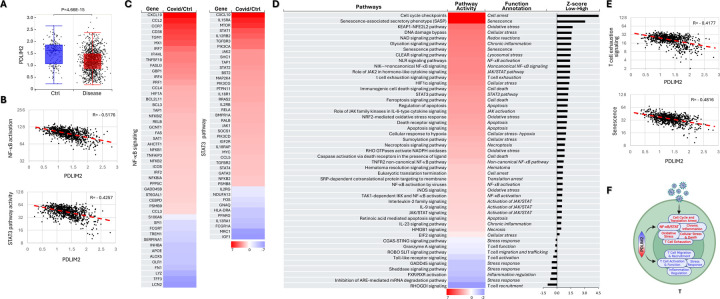
Association of PDLIM2 repression with T-cell exhaustion and senescence. (A) scRNA-seq data analysis showing PDLIM2 repression in the lung T cells of COVID-19 patients. P-value was obtained from Student’s *t* test. (B) Gene and pathway correlation showing negative associations of PDLIM2 expression with activations of the NF-κB and STAT3 signaling pathways in human lung T cells. (C) Heatmap showing differential expression of NF-κB and STAT3 target genes in the lung T cells of COVID-19 patients in comparison to uninfected controls. (D) Heatmap showing differential activation of signaling pathways in PDLIM2-low vs. -high expressing lung T cells in COVID-19 patients. (E) Gene and pathway correlation showing negative associations of PDLIM2 expression with the activation of the T-cell exhaustion and senescence signaling pathways in human lung T cells. (F) Cartoon summary listing the main signaling pathways differentially activated in PDLIM2-low vs. -high expressing lung T cells in COVID-19 patients.
