# Supplementary material for: PDLIM2 Repression: A Common Mechanism in Viral Lung Infection
Source: bioRxiv. 2025 Sep 17:2025.09.12.675949. Preprint. [Version 2] doi: 10.1101/2025.09.12.675949 (PMC12458227; doi:10.1101/2025.09.12.675949)
Supplement: 1 — Fig. S1: Top pathways altered in patient neutrophils. Fig. S2: Top pathways altered in patient monocytes. Fig. S3: Top pathways altered in patient macrophages. Fig. S4: Top pathways altered in patient T cells. Fig. S5: Top pathways altered in patient AT2 cells. Fig. S6: Top pathways altered in patient AT1 cells. Fig. S7: Top pathways altered in patient airway epithelial cells. Fig. S8: Top pathways altered in patient fibroblasts. Table S1: List of GEO datasets analyzed in the paper. Table S2: Main differences of neutrophil-related pathways and biological processes in patients with mild and severe conditions by lung viral infection. Table S3: Main differences of monocyte-related pathways and biological processes in patients with mild and severe conditions by lung viral infection. Table S4: Main differences of macrophage-related pathways and biological processes in patients with mild and severe conditions by lung viral infection. Table S5: Main differences of T-cell-related pathways and biological processes in patients with mild and severe conditions by lung viral infection. Table S6: Main differences of AT2 cell-related pathways and biological processes in patients with mild and severe conditions by lung viral infection. Table S7: Main differences of AT1 cell-related pathways and biological processes in patients with mild and severe conditions by lung viral infection. Table S8: Main differences of AEC-related pathways and biological processes in patients with mild and severe conditions by lung viral infection. Table S9: Main differences of lung fibroblast-related pathways and biological processes in patients with mild and severe conditions by lung viral infection. [file NIHPP2025.09.12.675949V2-supplement-1.pdf]

Fig. S1_Neutrophil

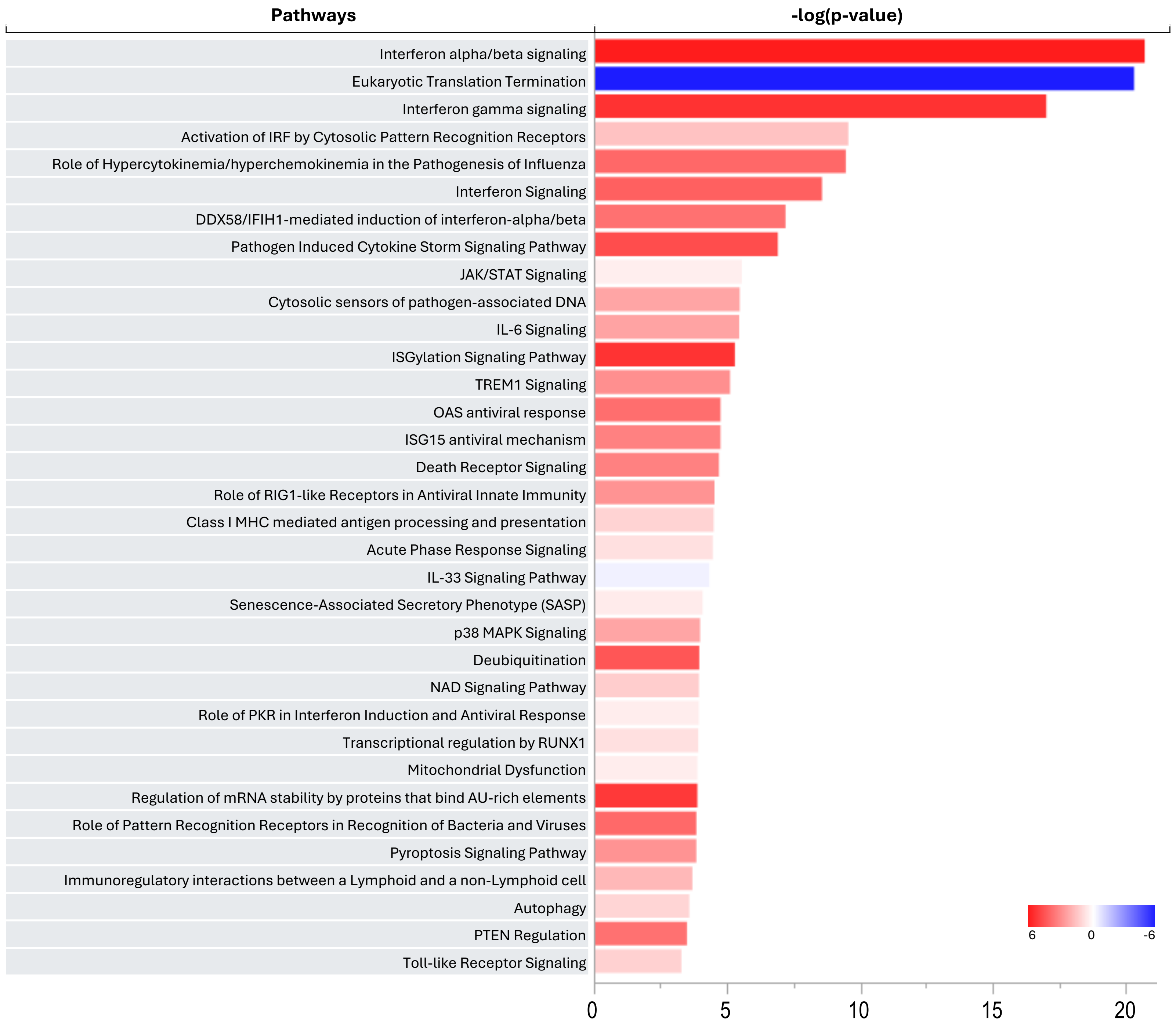

Fig. S2_Monocyte

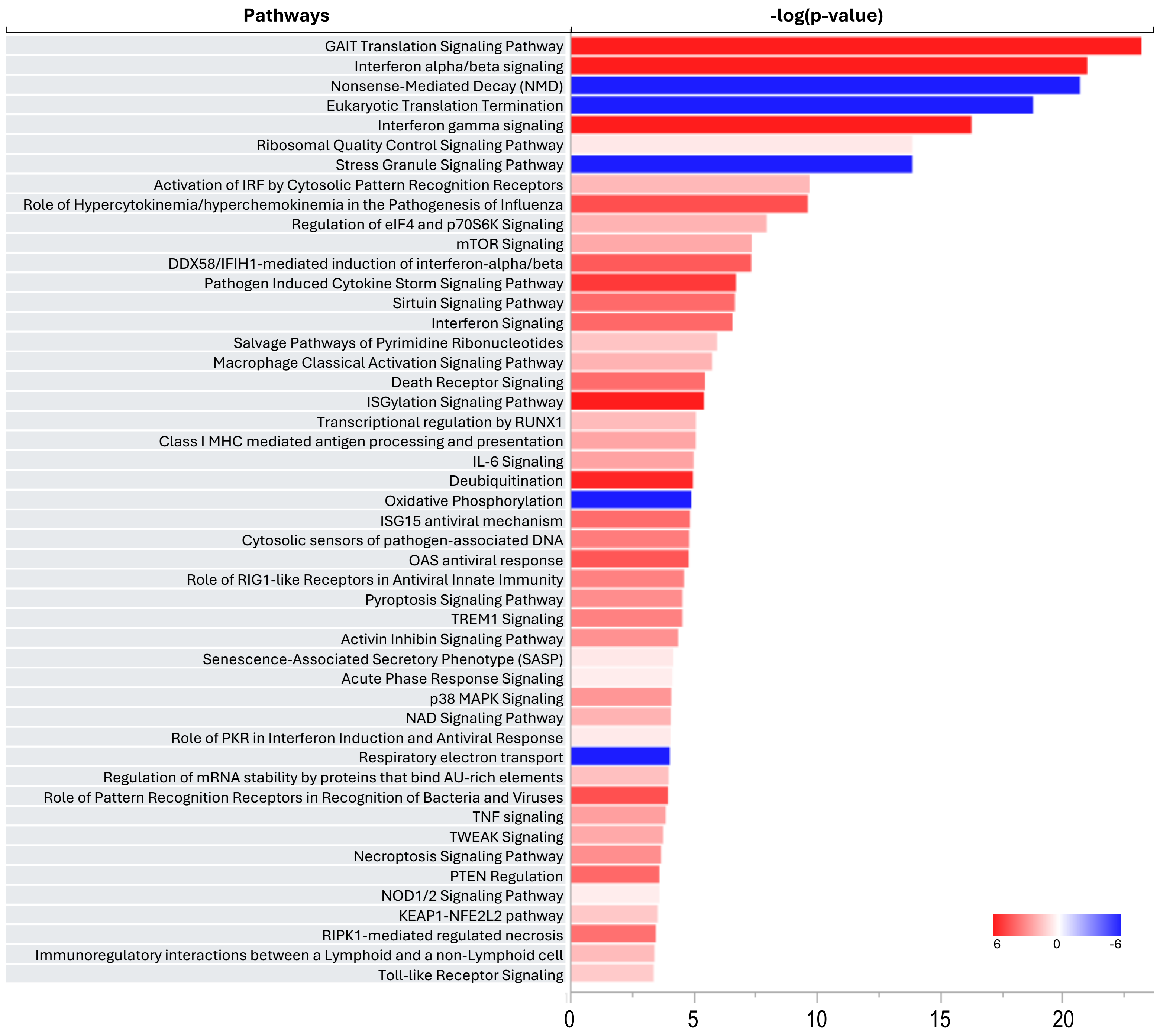

Fig. S3_Macrophage

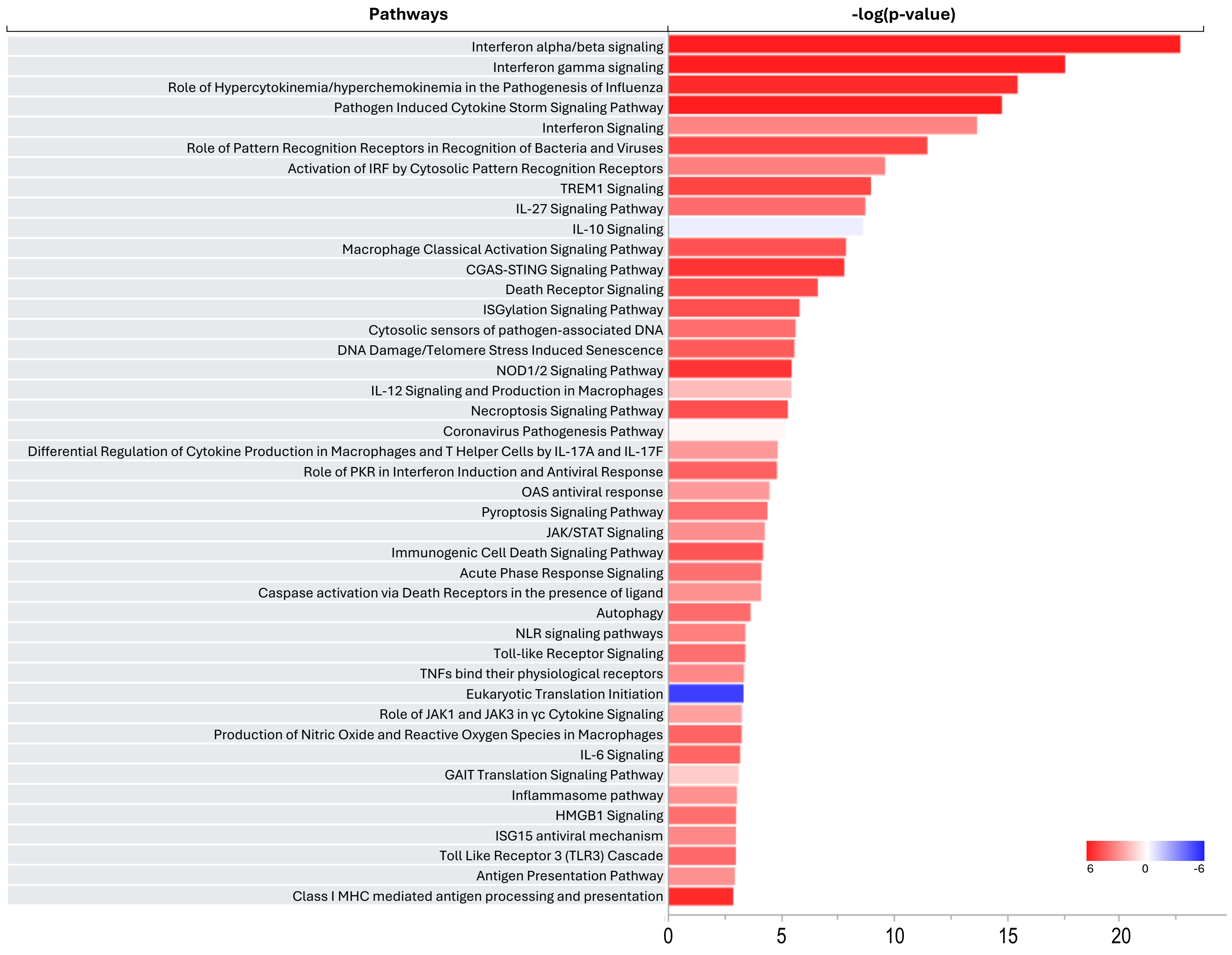

Fig. S4_T Cell

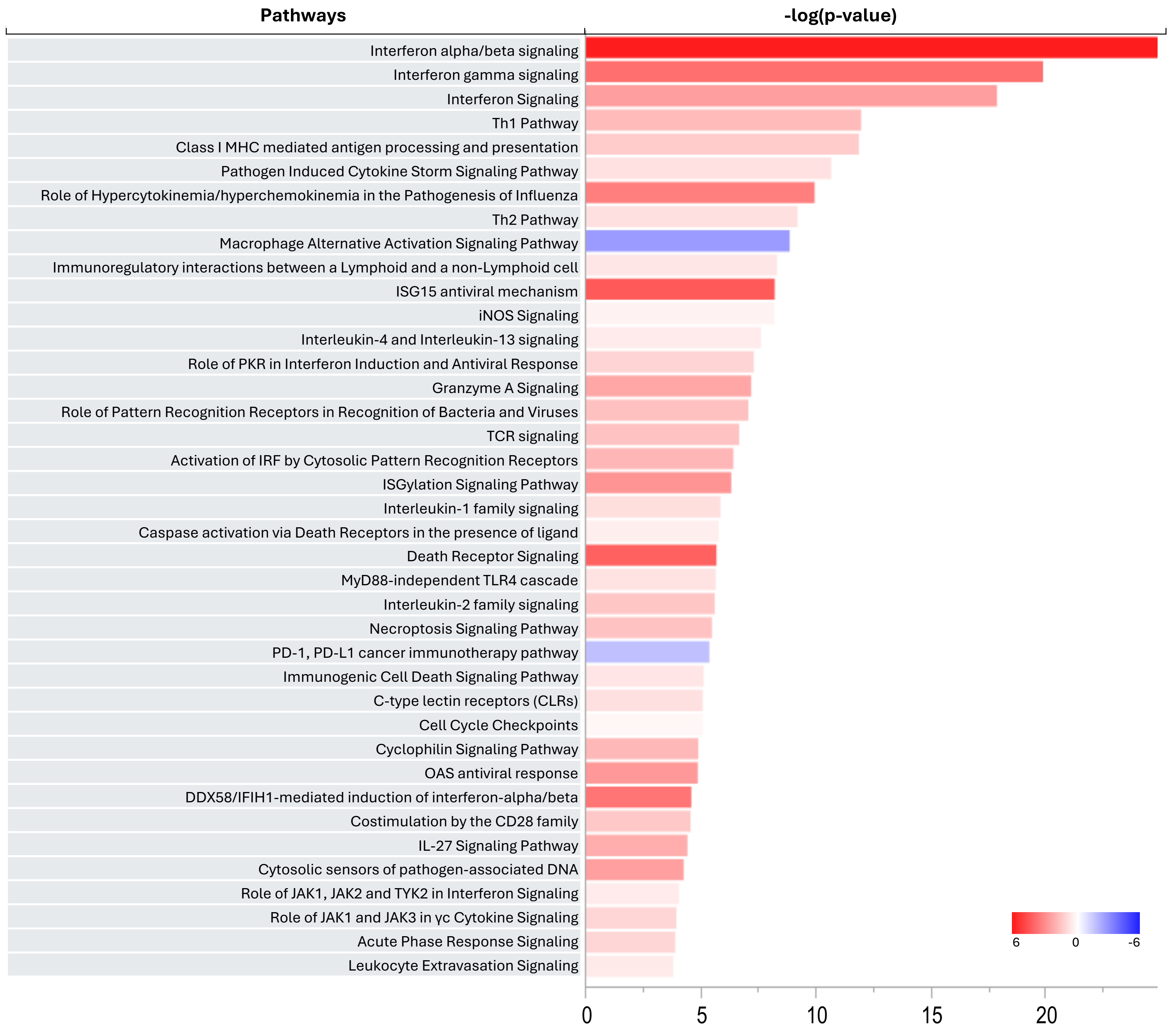
Pathways
-log(p-value)
Interferon alpha/beta signaling
Interferon gamma signaling
Interferon Signaling
Th1 Pathway
Class I MHC mediated antigen processing and presentation
Pathogen Induced Cytokine Storm Signaling Pathway
Role of Hypercytokinemia/hyperchemokinemia in the Pathogenesis of Influenza
Th2 Pathway
Macrophage Alternative Activation Signaling Pathway
Immunoregulatory interactions between a Lymphoid and a non-Lymphoid cell
ISG15 antiviral mechanism
iNOS Signaling
Interleukin-4 and Interleukin-13 signaling
Role of PKR in Interferon Induction and Antiviral Response
Granzyme A Signaling
Role of Pattern Recognition Receptors in Recognition of Bacteria and Viruses
TCR signaling
Activation of IRF by Cytosolic Pattern Recognition Receptors
ISGylation Signaling Pathway
Interleukin-1 family signaling
Caspase activation via Death Receptors in the presence of ligand
Death Receptor Signaling
MyD88-independent TLR4 cascade
Interleukin-2 family signaling
Necroptosis Signaling Pathway
PD-1, PD-L1 cancer immunotherapy pathway
Immunogenic Cell Death Signaling Pathway
C-type lectin receptors (CLRs)
Cell Cycle Checkpoints
Cyclophilin Signaling Pathway
OAS antiviral response
DDX58/IFIH1-mediated induction of interferon-alpha/beta
Costimulation by the CD28 family
IL-27 Signaling Pathway
Cytosolic sensors of pathogen-associated DNA
Role of JAK1, JAK2 and TYK2 in Interferon Signaling
Role of JAK1 and JAK3 in γc Cytokine Signaling
Acute Phase Response Signaling
Leukocyte Extravasation Signaling
6
0
-6
0
5
10
15
20

Fig. S5_AT2

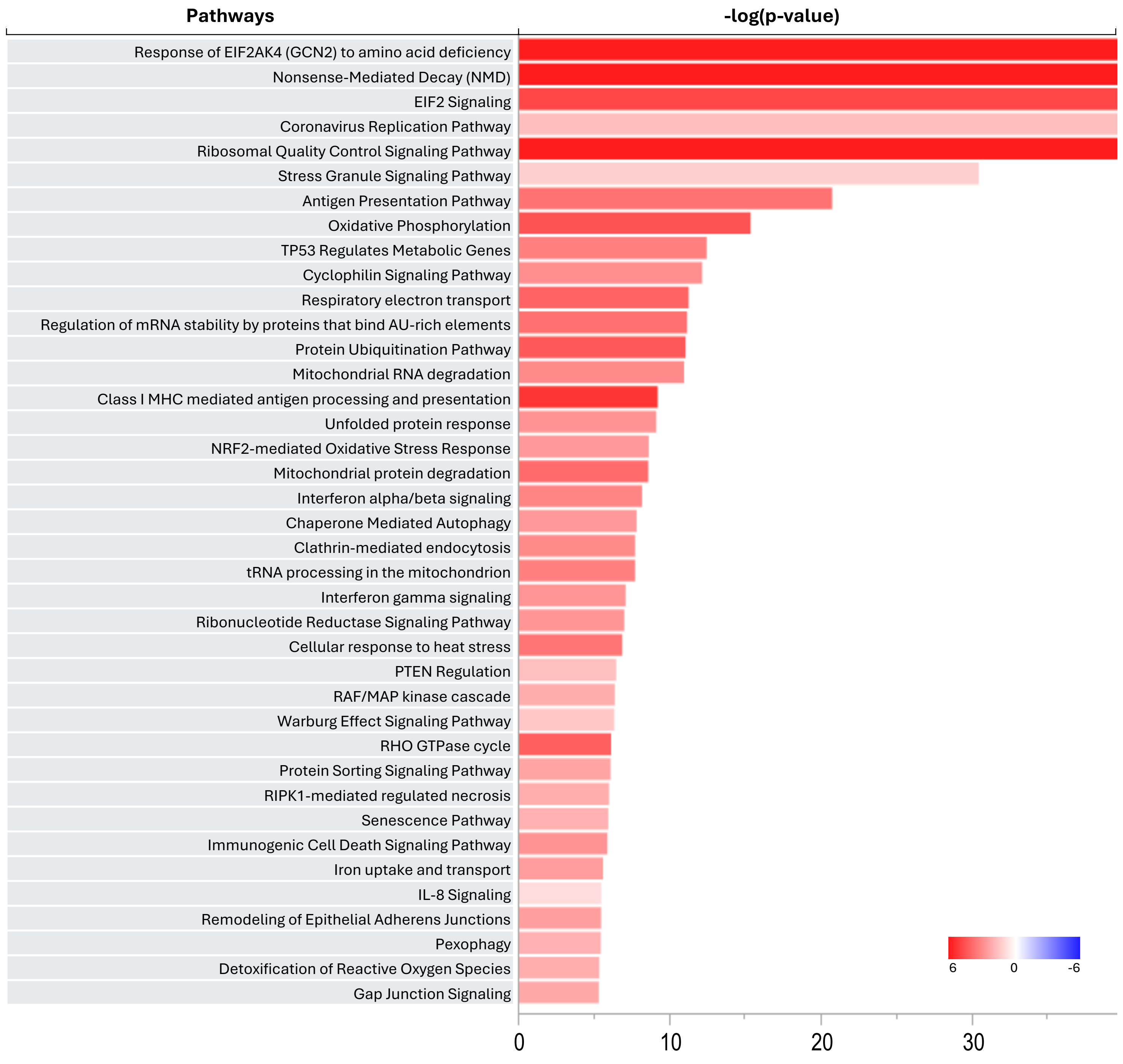
Pathways
-log(p-value)
Response of EIF2AK4 (GCN2) to amino acid deficiency
Nonsense-Mediated Decay (NMD)
EIF2 Signaling
Coronavirus Replication Pathway
Ribosomal Quality Control Signaling Pathway
Stress Granule Signaling Pathway
Antigen Presentation Pathway
Oxidative Phosphorylation
TP53 Regulates Metabolic Genes
Cyclophilin Signaling Pathway
Respiratory electron transport
Regulation of mRNA stability by proteins that bind AU-rich elements
Protein Ubiquitination Pathway
Mitochondrial RNA degradation
Class I MHC mediated antigen processing and presentation
Unfolded protein response
NRF2-mediated Oxidative Stress Response
Mitochondrial protein degradation
Interferon alpha/beta signaling
Chaperone Mediated Autophagy
Clathrin-mediated endocytosis
tRNA processing in the mitochondrion
Interferon gamma signaling
Ribonucleotide Reductase Signaling Pathway
Cellular response to heat stress
PTEN Regulation
RAF/MAP kinase cascade
Warburg Effect Signaling Pathway
RHO GTPase cycle
Protein Sorting Signaling Pathway
RIPK1-mediated regulated necrosis
Senescence Pathway
Immunogenic Cell Death Signaling Pathway
Iron uptake and transport
IL-8 Signaling
Remodeling of Epithelial Adherens Junctions
Pexophagy
Detoxification of Reactive Oxygen Species
Gap Junction Signaling
6
0
-6
0
10
20
30

Fig. S6_AT1

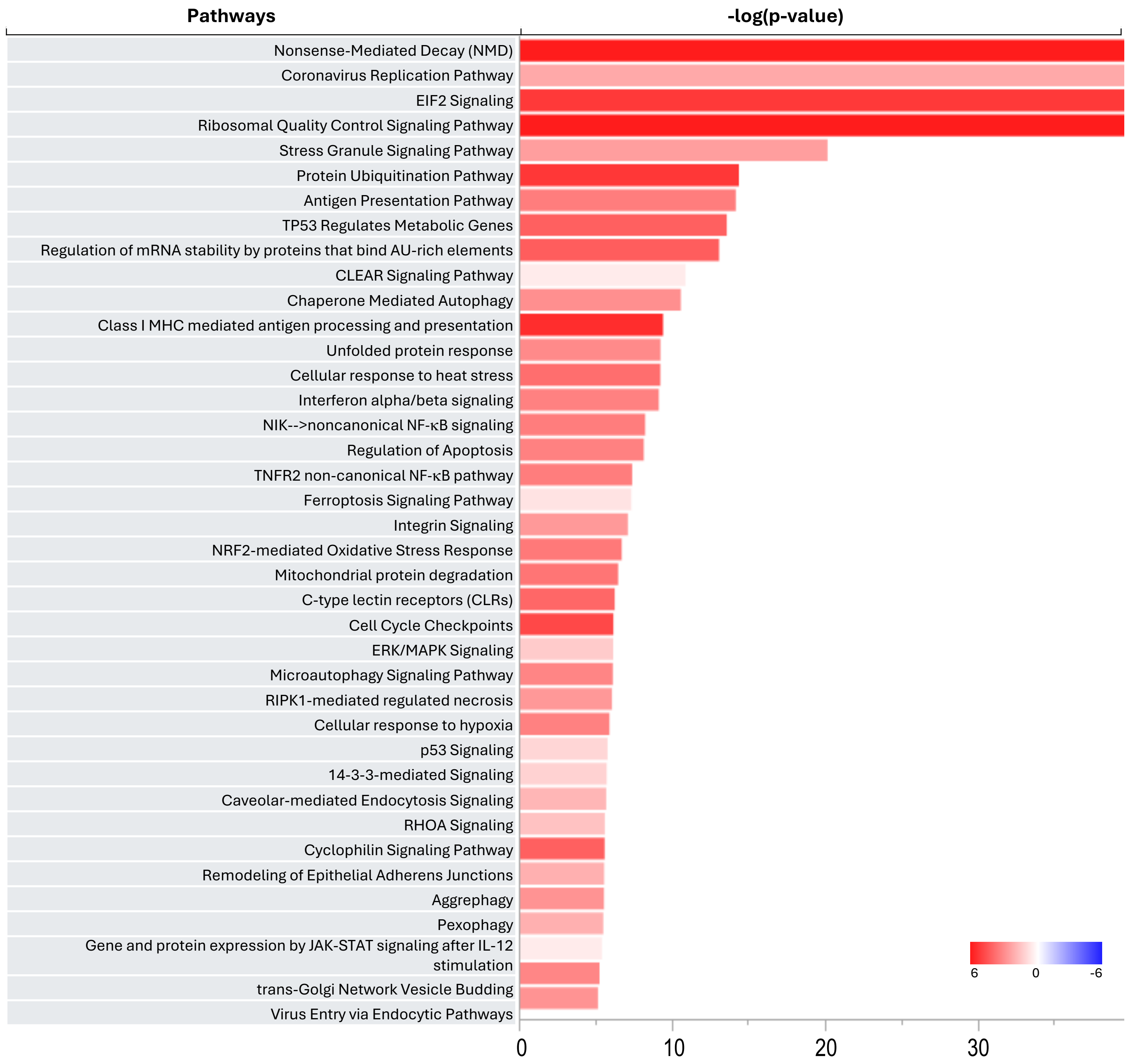
Pathways
-log(p-value)
Nonsense-Mediated Decay (NMD)
Coronavirus Replication Pathway
EIF2 Signaling
Ribosomal Quality Control Signaling Pathway
Stress Granule Signaling Pathway
Protein Ubiquitination Pathway
Antigen Presentation Pathway
TP53 Regulates Metabolic Genes
Regulation of mRNA stability by proteins that bind AU-rich elements
CLEAR Signaling Pathway
Chaperone Mediated Autophagy
Class I MHC mediated antigen processing and presentation
Unfolded protein response
Cellular response to heat stress
Interferon alpha/beta signaling
NIK-->noncanonical NF-κB signaling
Regulation of Apoptosis
TNFR2 non-canonical NF-κB pathway
Ferroptosis Signaling Pathway
Integrin Signaling
NRF2-mediated Oxidative Stress Response
Mitochondrial protein degradation
C-type lectin receptors (CLRs)
Cell Cycle Checkpoints
ERK/MAPK Signaling
Microautophagy Signaling Pathway
RIPK1-mediated regulated necrosis
Cellular response to hypoxia
p53 Signaling
14-3-3-mediated Signaling
Caveolar-mediated Endocytosis Signaling
RHOA Signaling
Cyclophilin Signaling Pathway
Remodeling of Epithelial Adherens Junctions
Aggrephagy
Pexophagy
Gene and protein expression by JAK-STAT signaling after IL-12 stimulation
trans-Golgi Network Vesicle Budding
Virus Entry via Endocytic Pathways
0
10
20
30
6
0
-6

Fig. S7_Airway Epithelium

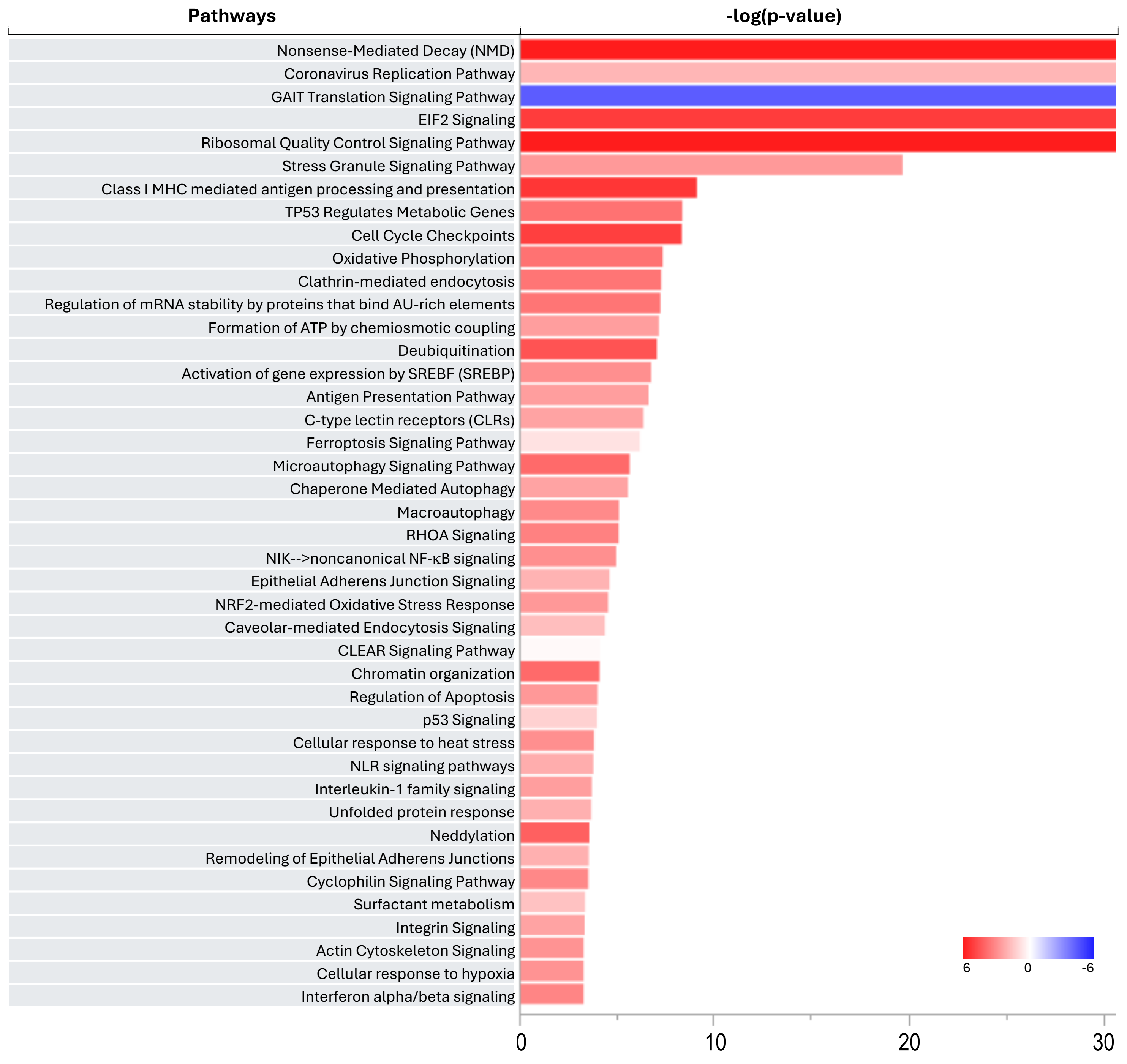

Fig. S8_Fibroblast

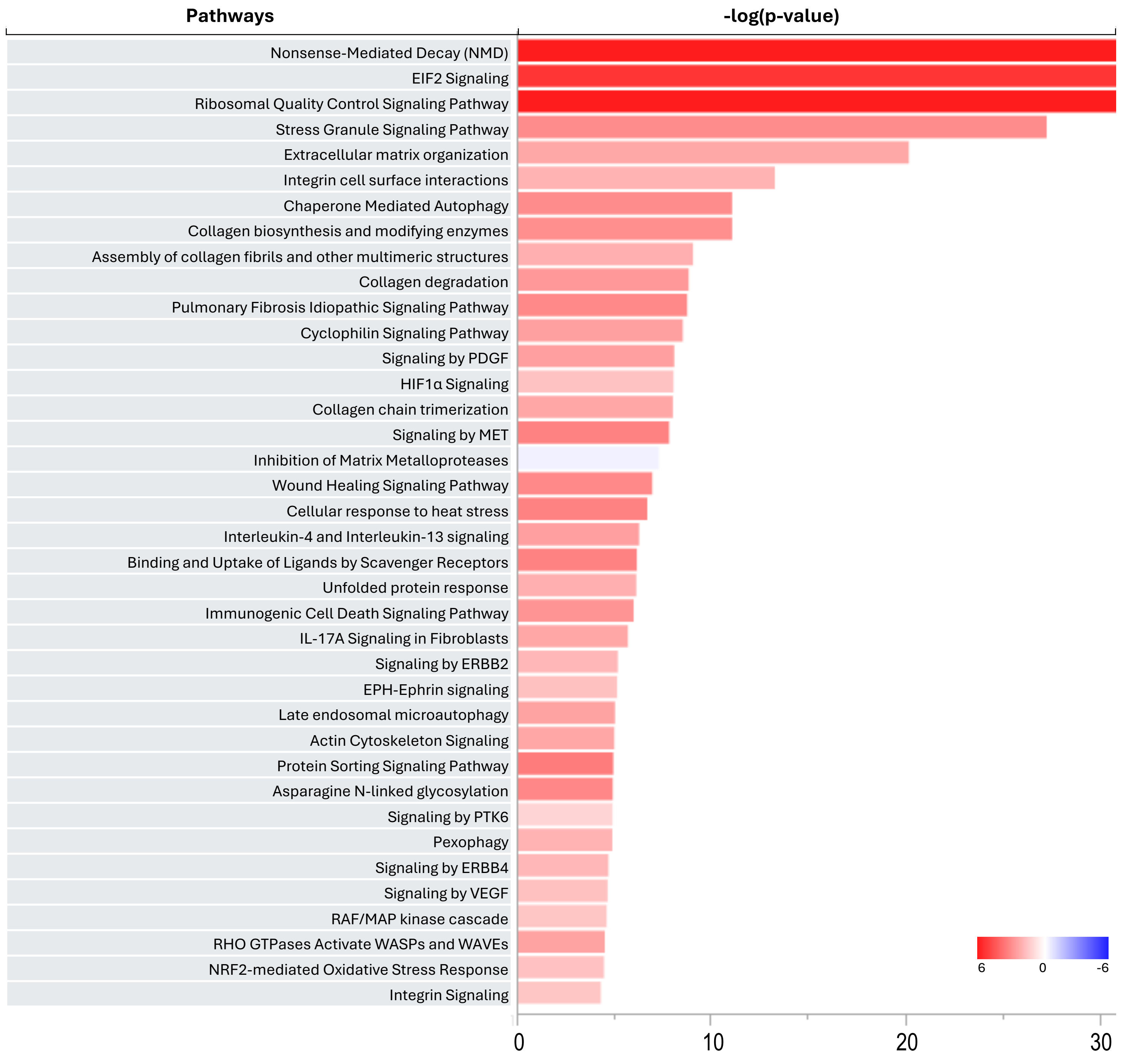

Table S1: List of GEO datasets analyzed in the paper.

GEO	Data	Sample	Virus Type	R scripts on GitHub	Figures in Paper	References
GSE101702	Microarray	Blood	Influenza	GSE101702	Fig.1A	Tang et al., 2019 (PMID:31366921)<br>Zerbib et al., 2020 (PMID: 32066441)
GSE157103	Bulk RNAseq	Blood	SARS-CoV-2	GSE157103	Fig.1B	Overmyer et al., 2021 (PMID: 33096026)
GSE243629	scRNAseq	Blood	Influenza	GSE243629	Fig.2&3	Zhang et al., 2023 (PMID: 38089584)
GSE145926	scRNAseq	BAL	SARS-CoV-2	GSE145926	Fig.4&5	Liao et al., 2020 (PMID: 32398875)<br>Zhang et al., 2024 (PMID: 39369126)
GSE171524	scRNAseq	Lung Tissue	SARS-CoV-2	GSE171524	Fig.6,7,8,9	Melms et al., 2021 (PMID: 33915568)

Table S2: Main differences of neutrophil-related pathways and biological processes in patients with mild and severe conditions by lung viral infection.

Category	Severe	Mild	Pathways and Biological Processes Involved
Neutrophilia	A characteristic feature of severe infection is neutrophilia. This is often accompanied by lymphopenia, leading to a high neutrophil-to-lymphocyte ratio (NLR), a strong predictor of disease severity and poor outcomes. This sustained high count indicates an ongoing, uncontrolled inflammatory process and emergency granulopoiesis.	While some increase in neutrophil count might occur, it is generally less pronounced and more transient compared to severe cases, returning to normal levels as the infection resolves.	1. Neutrophilia<br>2. Excessive and uncontrolled formation of NETs (NETosis)<br>3. “Emergency granulopoiesis“-degranulation<br>4. Impaired phagocytosis<br>5. Cytokine storm and systemic inflammation<br>6. Thrombosis<br>7. Cell stress and immunogenic death
Neutrophil Activation and Phenotype	Highly activated but with an altered, pro-inflammatory, and sometimes "immature" phenotype: increased activation markers; dysfunctional responses (impaired phagocytosis); reprogramming into polymorphonuclear myeloid-derived suppressor cells (PMN-MDSCs); and altered metabolism (GAPDH inhibition, etc.).	More balanced activation and generally more effective functions in clearing the virus without excessive bystander damage; less likely to adopt the highly inflammatory or immunosuppressive phenotypes seen in severe cases.	
Neutrophil Extracellular Traps	Excessive and uncontrolled formation of NETs (NETosis): pro-thrombotic effects; tissue damage; amplified inflammation; impaired NET clearance.	The production is more controlled, and the body's mechanisms for clearing NETs are generally effective, preventing widespread immunothrombosis and tissue damage.	
Contribution to Cytokine Storm	Significant productions of pro-inflammatory cytokines and chemokines (e.g., IL-6, IL-8, TNF-α, MCP-1), further fueling the cytokine storm that drives systemic inflammation and multi-organ dysfunction.	Neutrophils contribute to inflammation in a regulated manner, with a balanced response to resolve the infection.	

Table S3: Main differences of monocyte-related pathways and biological processes in patients with mild and severe conditions by lung viral infection

Category	Severe	Mild	Pathways and Biological Processes Involved
Cell numbers and subsets	Often high monocytosis in the peripheral blood, with a particular increase in inflammatory monocytes and lung recruitment.	While monocyte counts may increase during acute infection, the overall increase is typically less pronounced. Monocyte subsets are more balanced, and lung recruitment is more controlled.	1. Monocytosis<br>2. High monocyte recruitment<br>3. Inflammasome activation<br>4. Hyper-inflammation<br>5. Cell stress and immunogenic cell death<br>6. Increased antigen presentation
Activation and pro-inflammatory cytokine production	High production of pro-inflammatory cytokines and chemokines: TNFα, IL-6, type I IFNs, MCP-1 (CCL2), MIP-1α (CCL3), and IP-10 (CXCL10); and hyper inflammasome activation (e.g., NLRP3 activation for IL-1 and IL-18 release) and pyroptosis.	More regulated and self-limiting. The cytokine production is proportionate to the viral load and contributes to effective viral clearance without causing excessive systemic inflammation.	
Antigen presentation	Downregulation of HLA-DR leading to impaired antigen presentation and T cell activation (immunoparalysis)	Normal or even upregulated HLA-DR expression	
Differentiation and role in lung pathology	The inflammatory monocytes recruited to the lungs differentiate into highly pro-inflammatory macrophages and dendritic cells. These cells contribute significantly to lung injury and ARDS by: direct tissue damage; exacerbating inflammation; promoting fibrosis; secondary infections.	Monocytes effectively differentiate into macrophages and dendritic cells that contribute to viral clearance and then change to a reparative phenotype, facilitating tissue healing and inflammation resolution.	

Table S4: Main differences of macrophage-related pathways and biological processes in patients with mild and severe conditions by lung viral infection

Category	Severe	Mild	Pathways and Biological Processes Involved
Macrophage activation and phenotype (polarization)	Hyper pro-inflammatory M1 polarization and state; increased inflammasome activation (e.g., NLRP3 inflammasome); impaired inflammation resolution activity; dysfunctional efferocytosis; and altered surface markers (increased CD163).	Controlled inflammation; effective viral clearance; and balanced polarization.	1. M1/M2 macrophage<br>2. Phagocytosis and efferocytosis<br>3. Cytokine storm<br>4. Production of nitric oxide and reactive oxygen species in macrophages<br>5. Inflammasome activation<br>6. The systemic inflammatory response syndrome (SIRS)<br>7. The increased inflammatory monocyte-derived macrophages particularly in the lung<br>8. Cell death
Cytokine production	Profound and sustained production of pro-inflammatory cytokines and chemokines (e.g., IL-1β, IL-6, TNF-α, MCP-1, MIP-1α, IP-10, etc.), leading to the systemic inflammatory response syndrome (SIRS) and potential multi-organ dysfunction.	More transient and localized production of cytokines, sufficient to control the infection without causing widespread hyper-inflammation.	
Recruitment and accumulation	Increased recruitment and accumulation of inflammatory monocytes and differentiation into macrophages, particularly in the lung.	While macrophages are recruited to the infection sites, their numbers and activity are more balanced.	
Summary	The dysregulation and sustained pro-inflammatory activation of macrophages lead to a runaway inflammatory process that damages host tissues and is a major driver of pathology	A controlled, immune response that resolves inflammation after viral clearance	

Table S5: Main differences of T-cell-related pathways and biological processes in patients with mild and severe conditions by lung viral infection

Category	Severe	Mild	Pathways and Biological Processes Involved
Lymphopenia	A hallmark of severe infectious diseases like COVID-19 is pronounced lymphopenia, particularly CD4+ helper and CD8+ cytotoxic T cells: redistribution/sequestration; apoptosis/pyroptosis; bone marrow suppression; and T cell exhaustion.	While some degree of transient lymphopenia may occur, it is generally less severe, and T-cell counts tend to recover more quickly compared to severe cases.	1. Lymphopenia<br>2. T cell exhaustion<br>3. Hyperinflammation<br>4. Cytokine storm<br>5. Cell stress<br>6. Senescence<br>7. Apoptosis/pyroptosis
T cell exhaustion	Functionally impaired due to prolonged and intense antigen stimulation and exposure to high levels of inflammatory cytokines	T cells generally maintain better functionality and are less prone to severe exhaustion.	
Virus-specific T cell response	Patients with severe COVID-19 may show a robust and sometimes even stronger sars-cov-2-specific T cell response in terms of magnitude. However, the response is ineffective due to lymphopenia and T-cell exhaustion, leading to hyperinflammation rather than effective viral clearance.	Patients typically develop a more effective and well-coordinated sars-cov-2-specific T cell response. They often have better memory T-cell responses that contribute to long-term immunity.	
Cytokine production	The overall production of key antiviral cytokines like IFN-γ by T cells might be lower, however, many other inflammatory cytokines are upregulated.	T cells produce appropriate levels of antiviral cytokines, particularly IFN-γ	
T cell subsets	Expansion of highly differentiated T cells (e.g., terminal effector memory T cells re-expressing CD45RA), a sign of chronic immune activation and exhaustion.	A more balanced T cell subset distribution, with effective memory T cell populations that contribute to rapid responses upon re-exposure.	

Table S6: Main differences of AT2 cell-related pathways and biological processes in patients with mild and severe conditions by lung viral infection

Category	Severe	Mild	Pathways and biological processes involved
Viral infection and replication	Extensive and sustained virus infection and replication within AT2 cells, leading to direct viral cytotoxicity, high viral load (persistent inflammation), senescence-associated secretory phenotype (SASP), and pro-inflammatory phenotype (cytokine storm and chronic inflammation).	While AT2 cells are infected, the viral load is generally lower, and the infection is more effectively controlled by the early immune response. The extent of direct viral damage and cell death is less widespread, allowing for more effective recovery.	1. Higher infection<br>2. Hyperinflammation, cytokine storm<br>3. Cell death and senescence<br>4. Surfactant dysfunction<br>5. Compromised antiviral interferon response<br>6. Impaired regeneration (proliferation and differentiation)<br>7. Fibrosis promotion
Surfactant production	Reduced surfactant production, surfactant dysfunction, and alveolar collapse (atelectasis).	Surfactant production may be transiently affected, no widespread surfactant deficiency and subsequent alveolar collapse.	
Role in lung repair and regeneration	Impaired proliferation and differentiation, aberrant differentiation/maladaptive repair, and fibrosis promotion.	Effective proliferation and differentiation, resolution of inflammation	
Immunomodulatory role	Dysfunctional AT2 cells contribute to the cytokine storm by releasing pro-inflammatory mediators. Their impaired function can also lead to a less effective early antiviral interferon response, potentially allowing for more viral replication and exacerbating the immune-mediated damage	AT2 cells contribute to a more balanced innate immune response, producing interferons and other mediators that help control viral replication without triggering overwhelming inflammation.	

Table S7: Main differences of AT1 cell-related pathways and biological processes in patients with mild and severe conditions by lung viral infection

Category	Severe	Mild	Pathways and biological processes involved
Direct damage and cell death	High damage and death (pyroptosis and apoptosis) by the overwhelming infection and inflammatory response, including cytokine storm, neutrophil extracellular traps (nets), oxidative stress; and hyaline membrane formation.	The damage is generally localized and less widespread. The inflammatory response is more controlled, limiting bystander injury to AT1 cells.	1. Viral infection<br>2. High production of inflammation cytokines and chemokines/Cytokine storm<br>3. Cellular stress: oxidative, inflammation, hypoxemia, etc.<br>4. Cell damage and death: apoptosis, pyroptosis, etc.<br>5. Pro-fibrotic environment<br>6. ARDS<br>7. Excessive and uncontrolled recruitment of inflammatory cells
Impaired repair and regeneration	Defects in AT1 cell differentiation from infected or highly stressed AT2 cells or pause at the "transitional state" of AT2 to AT1 cells.	AT2 cells effectively proliferate and differentiate into functional AT1 cells, ensuring efficient repair of the alveolar epithelium.	
Functional consequences	Severe impairment of gas exchange; acute respiratory distress syndrome (ARDS); persistent hypoxemia; and long-term lung damage.	Minimal impact on gas exchange due to limited and transient AT1 cell damage and effective repair.	
Immunomodulatory role	Sustained release of cytokines and chemokines; excessive and uncontrolled recruitment of inflammatory cells; and widespread death and the loss of the epithelial barrier.	Controlled and localized immune response from their cytokine and chemokine production; and the integrity of AT1 layer acting as a barrier for pathogens and immune cells.	

Table S8: Main differences of AEC-related pathways and biological processes in patients with mild and severe conditions by lung viral infection

<table>
<thead>
<tr><th>Category</th><th>Severe</th><th>Mild</th><th>Pathways and Biological Processes Involved</th></tr>
</thead>
<tbody>
<tr><td>Viral infection and spread</td><td>Extensive viral infection and deep spread; and high viral loads</td><td>The initial infection might be more localized, often starting in the upper respiratory tract (nasal mucosa).</td><td rowspan="4">1. Viral infection and spread
2. Hyper inflammatory, exacerbated/systemic cytokine/chemokine storm
3. Class I MHC mediated antigen processing and presentation
4. IFN response, pattern recognition
5. Immune cell infiltration
6. Severe cell stress, damage, injury, death
7. Impaired regeneration/repair</td></tr>
<tr><td>Antiviral response</td><td>Dysregulated antiviral response; delayed/suppressed IFN response; hyperinflammation; immune cell infiltration; and bystander damage.</td><td>More effective and controlled early antiviral IFN response with timely IFN production; controlled pro-inflammatory cytokines and chemokines release</td></tr>
<tr><td>Cellular damage and repair</td><td>Severe cellular damage and impaired repair; widespread cell injury and death; hyaline membrane formation; loss of barrier integrity; disrupted mucociliary clearance; and impaired regeneration and fibrosis</td><td>Transient cilia shedding and mucociliary dysfunction; limited programmed cell death (necroptosis); preserved epithelial repair and regeneration</td></tr>
<tr><td>Immunomodulatory roles</td><td>Impaired pattern recognition and early warning; immunomodulatory role becomes maladaptive and contributes to pathogenesis: delayed or dysregulated immune response, exacerbated cytokine/chemokine storm.</td><td>Effective pattern recognition and early warning; and controlled and proportionate production of IFNs and pro-inflammatory cytokines/chemokines, restricting viral replication and shaping immunity .</td></tr>
</tbody>
</table>

Table S9: Main differences of lung fibroblast-related pathways and biological processes in patients with mild and severe conditions by lung viral infection

Category	Severe	Mild	Pathways and Biological Processes Involved
Fibroblast activation and differentiation	Persistent and dysregulated activation; high levels of pro-inflammatory cytokines e.g., IL-6, TNF-α) and growth factors, especially transforming growth factor-beta (TGF-β), are crucial drivers; and epithelial-mesenchymal transition (EMT).	Fibroblasts are activated in response to limited epithelial cell damage and localized inflammation.	1. Excessive collagen/ECM deposition, fibrosis<br>2. Fibroblast activation and differentiation<br>3. Myofibroblast persistence<br>4. Production of inflammatory cytokines/chemokines and TGF-β<br>5. Recruitment of inflammatory immune cells<br>6. Class I MHC mediated antigen processing and presentation<br>7. Interferon alpha/beta signaling
Myofibroblast	Pathological myofibroblast persistence; and resistant to apoptosis and persist in the lung.	Typically undergo apoptosis (programmed cell death) or dedifferentiate back into quiescent fibroblasts.	
Extracellular matrix (ECM)	Excessive ECM deposition and scarring; massive collagen deposition; architectural distortion; and fibrotic foci formation	The production and deposition of ECM are tightly controlled and regulated.	
Cross-talk with immune cells	Engage in a vicious cycle with immune cells: produce chemokines that recruit more inflammatory cells; and influence the phenotype and function of immune cells, potentially promoting a pro-fibrotic immune response.	A more controlled and regulated communication with immune cells.	
